# The Effect of the Quantity and Distribution of Teammates’ Tendency Toward Self-Interest and Altruism on Individual Decision-Making

**DOI:** 10.3389/fpsyg.2021.785806

**Published:** 2022-02-11

**Authors:** Mi Zou, Jinqiu Feng, Nan Qin, Jiangdong Diao, Yang Yang, Jiejie Liao, Jiabao Lin, Lei Mo

**Affiliations:** ^1^Key Laboratory of Brain, Cognition and Education Sciences, South China Normal University, Ministry of Education, Guangzhou, China; ^2^School of Psychology, South China Normal University, Guangzhou, China; ^3^School of Psychology, Center for Studies of Psychological Application, Guangzhou, China; ^4^School of Psychology, Guangdong Key Laboratory of Mental Health and Cognitive Science, Guangzhou, China

**Keywords:** altruistic tendency, self-interest tendency, cooperation, credibility, decentralized effect, convergent effect

## Abstract

Previous studies have explored the impact of the cost ratio of individual solutions versus collective solutions on people’s cooperation tendency in the presence of individual solutions. This study further explored the impact of team credibility on people’s propensity to cooperate in the presence of individual solutions. Study 1 investigated the influence of different level of altruistic tendencies or the self-interest tendencies of teammates on participants’ decision-making. Study 2 explored the influence of the distribution of altruistic tendencies or self-interest tendencies on participants’ decision-making. The results of Study 1 showed that the proportion of participants who chose the collective solution increased with an increase in the altruistic tendencies of the team. When the altruistic tendencies of the teammates reached a certain value, the proportion of participants taking the collective solution showed a trend to stabilize. Furthermore, the proportion of participants who chose the individual solution increased with the increase in the self-interest tendencies of the team. When the self-interest tendencies of the teammates reached a certain value, the individual solution was stably adopted. The results of Study 2 showed that with the total altruistic tendency remaining unchanged, the more altruistic group members that altruistic tendencies were allocated to, the higher a participant’s level of trust in the team would be, which showed the decentralized effect of altruistic tendencies. In the case that the total self-interest tendency was unchanged, the fewer self-interest group members the self-interest tendencies were allocated to, the higher a participant’s level of trust in the team would be, which showed the convergent effect of self-interest tendencies.

## Introduction

Since ancient times, cooperation has played an important role in the struggle for human survival. The solutions for common problems such as climate change, resource reduction and environmental pollution require cooperation behaviors (Dietz et al., 2003; Fehr and Fischbacher, 2003; Milinski et al., 2006, 2008; Hauser et al., 2014). Cooperation, such as public education and public transportation, provides individual needs in a way that can maximize resource utilization. Cooperation exists at many levels and takes place between individuals and organizations as well as between states and countries. Cooperation allows participants to exchange valuable information that helps both sides improve their knowledge bases and work in a time- and resource-efficient manner (White, 2005).

In order to maintain and facilitate cooperation, abundant research has been conducted to explore influential factors (Cronk, 1999; White, 2005; Tabellini, 2008; Balliet, 2010; Espín et al., 2012; Gross et al., 2016; Molenmaker et al., 2016; Yilmaz and Bahçekapili, 2016) and underpinning mechanisms (Gardner and West, 2004; Fowler, 2005; Boyd et al., 2010; Wang et al., 2017; Fu et al., 2019) of cooperation. Specifically, there is by now considerable and convincing evidence that group members’ behavior within a social dilemma is influenced by both expectations and observations of others’ behavior (Braver and Barnett, 1974; Dawes et al., 1977; Messick et al., 1983; Schroeder et al., 1983; Yamagishi and Sato, 1986; Komorita et al., 1992; Bornstein and Ben-Yossef, 1994). There has also been much research conducted on the voluntary contributions mechanism (Brandts and Schram, 2001; Hauert et al., 2002; Juberías et al., 2004; Croson et al., 2005; Chaudhuri, 2011; García and Traulsen, 2012; Inglis et al., 2016; Iwamura et al., 2020), in which participants can choose between a private account and a public account to invest in; participants can obtain more profits from the public account if the collective investment of all the group members to the public account is high enough. Jennifer Zelmer (2003) performed a meta-analysis and concluded that average contributions, friendship among subjects, group size and cash reward are the main factors that have a significant effect on people’s tendencies to contribute to the public account. In the voluntary contributions game, subjects can benefit from both the private account and the public account.

However, few researchers have noted that society provides individual solutions as a complementary choice to shared problems. For example, a person can drive a private car to replace public transportation, or a house owner can employ private security instead of depending on a publicly funded police force. Theoretically, the availability of individual solutions leads to stronger tension than does the voluntary contributions game because people can only choose one of the two solutions (i.e., the individual solution or the collective solution) (Juberías et al., 2004; Gross and De Dreu, 2019). People try to obtain more independence through individual solutions, even if doing so consumes more resources (Fehr et al., 2002; Juberías et al., 2004). However, from existing theories, we know little about how individual solutions to shared problems influence the human inclination for cooperation and coordination.

Gross and De Dreu (2019) were the first to explore how the cost of an individual solution influences decision-making when the collective solution and the individual solution exist simultaneously; in their study, the researchers set different levels of cost ratios of an individual solution versus a collective solution and observed the subjects’ decision-making. The results showed that the participants’ cooperation tendencies increased with the increase in the cost ratio of the individual solution versus the collective solution. When the cost of the individual solution was over 1.7 times that of the collective solution, then 80% of participants would steadily choose the collective solution. Across different cost-benefit ratios of individually versus collectively solving the shared problem, individuals display a remarkable tendency toward group-independent, individual solutions. This “individualism” leads to inefficient resource allocations and coordination failure. In the presence of individual solutions to shared problems, groups struggle to balance self-reliance and collective efficiency, leading to a “modern tragedy of the commons.” In order to facilitate cooperation in this case, more research need to be done to know the underpinning mechanisms. We hold that partners’ cooperation tendency (i.e., participants’ judgment of the credibility of their partners in a cooperation activity) also plays an equal or more important role than that of cost ratio on participants’ cooperation tendencies in the presence of the individual solution.

Our research frame is divided into two parts. In the first part, we aimed to prove that team members’ credit is a significant factor which influences people’s cooperation tendencies when the individual solution is accessible. Then in the second part, we intended to investigate the exact nature of the relationship between others’ and our own behavior in a detailed way. A number of scholars have proposed uncooperative group members have greater impact on our behavior than equally-extreme cooperative ones. It has been reported (Messick et al., 1983; Samuelson et al., 1984) that providing a relatively wide distribution of false harvesting feedback in a resource-conservation dilemma leads to faster depletion of the shared resource than feedback with a narrow distribution and the same mean, just as one would expect if the extremely low cooperator had greater relative impact on others’ behavior. Furthermore, work by Kerr et al. (2009) has shown that the influence of a few non-cooperative group members is larger than the influence of a few cooperative group members (termed the “bad apple” effect). However, there has been no research exploring the influence of distribution of the self-interest or altruism tendencies on people’s decision making. Our current focus is on allocating the same self-interest or altruism tendencies to 2–5 group members, to explore how the distribution of self-interest or altruism tendencies influences people’s decisions.

To explore the question above, we designed the following four experiments to explore the effect of the quantity and distribution of teammates’ tendencies of self-interest and altruism on individual decision-making when an individual solution is available. First, the definition of relative items of this paper is given herein. The tendency of self-interest means that the group member contributes less than his or her fair share to the collective solution, while the tendency of altruism means that the group member contributes more than his or her fair share. In Study 1a and Study 1b, we explored whether one group member’s behavior can affect subjects’ decision-making. If one group member can influence subjects’ decision-making, we can infer that the credit of one’s partners is a basic factor in people’s individual or collective solution decision-making; furthermore, with more group members behaving in a similar way, the effect would be more significant. The hypothesis of Study 1a and Study 1b was that participants’ decision-making would be affected by one group member’s tendencies toward self-interest and altruism (i.e., that it would increase with an increase in the altruism tendencies and decrease with an increase in the self-interest tendencies). After we prove one group member’s behavior can influence participants’ individual or collective strategy decision-making in Study 1a and Study 1b, then, in Study 2a and Study 2b, we allocate the same self-interest or altruism tendencies to 2 to 5 group members. The hypothesis of Study 2a was that, with the total altruistic tendencies remaining unchanged, the more altruistic group members the altruistic tendencies were allocated to, the higher a participant’s level of trust in the team would be, while the hypothesis of Study 2b was that, with the total self-interest tendencies remaining unchanged, the fewer self-interest-based group members the self-interest tendencies were allocated to, the higher a participant’s level of trust in the team would be.

## Study 1A

According to previous research (Gross and De Dreu, 2019), when the cost ratio (i.e., cost ratio = the cost of the individual solution/the cost of the collective solution) increases to 1.75, people tend to choose the collective solution. In Study 1a, the cost ratio of the individual versus the collective solution was set to 1.75 as a “cooperation-oriented” situation. Because the cost of the collective solution is usually much lower than that of the individual solution in real life, we set the cooperation-oriented situation to better simulate reality.

Study 1a aimed to explore how the altruism and self-interest of one of the partners would influence people’s cooperation tendencies under the “cooperation-oriented” situation when an individual solution was available.

### Method

#### Participants

*A priori* power analysis, carried out using G*Power software (Faul et al., 2007), indicated that to detect a medium-effect size of *d* = 0.5, for the planned χ^2^ test, with an alpha of 0.05 and power = 0.80, a sample of 48 participants would be needed. Fifty-two undergraduate students from a major public university participated in Study 1a. Data from two participants were removed as they did not understand the experimental rules well, and data from two other participants were removed after they questioned the authenticity of the online experiment. This left us with a final sample of 48 participants (*M*_age_ = 19.29, *SD* = 1.11, 56.3% females). This research completed an ethical review. We confirm that all the research meets ethical guidelines and adheres to the legal requirements of the study country.

#### Procedure

##### Procedure Part 1

The first step was designed to convince the subjects of the authenticity of the online interaction experiment. The recruitment information for undergraduate students was published, and the participants were informed that the experiment was a large-scale online interactive experiment that was being conducted simultaneously at 6 universities. The subjects entered the registration interface by clicking the website link to fill in their basic registration information and select the nearest experimental site. The registration interface adopted the same background and format as those used in the formal experiment.

##### Procedure Part 2

**FIGURE 1:**
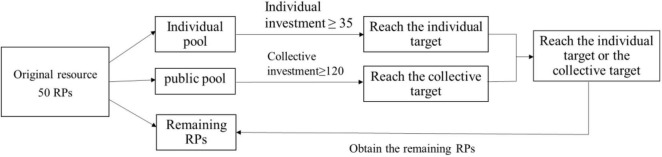
Experimental rules. The participants were confronted with a novel collective action problem in groups of six. Only when one of the individual targets and the collective target was reached could the participant obtain the remaining RPs.

Second, the subjects were seated alone in the lab in front of a computer screen to learn the rules of the game. In Study 1a, we confronted the participants with a novel collective action problem in a team of six members. Except for the participant, the other five members were virtual subjects set up by the computer program (i.e., for easier understanding, virtual group members set by computer programs were referred to “virtual subjects,” while people who took part in this experiment were referred to “participants”). Each member was endowed with 50 resource points (RPs). Members had to allocate their RPs to either their individual pool, a shared public pool, or keep any amount for themselves. The individual pool accepted only the individual investment of the participant, while the public pool accepted the joint investment of all six team members. Under the “cooperation-oriented” situation of Study 1a, a participant would keep the remaining resources that were not invested if the participant allocated enough resources to her individual pool to reach a predefined individual target (35 RPs). She would also keep her remaining resources if the group collectively allocated enough resources to the public pool to reach a predefined public target (120 RPs). If group members did not reach either their private target or the public target, then they lost everything (see Figure 1 as an illustration). Before carrying out the formal experiment, the participants had to pass the rule comprehension examination. Obviously, according to the rules of the cooperation-oriented game, each participant needed to allocate 35 RPs to the individual pool to obtain the remaining 15 RPs, while if the collective solution was adopted for the public target, each person only needed to allocate 20 RPs to obtain the remaining 30 RPs. The cost ratio of the individual solutions versus the collective solutions was 35:20, and the profit ratio of the individual solutions versus the collective solutions was 15:30. According to the results of a previous study (Gross and De Dreu, 2019), most subjects tend to adopt the collective solution in this circumstance.

##### Procedure Part 3

**FIGURE 2:**
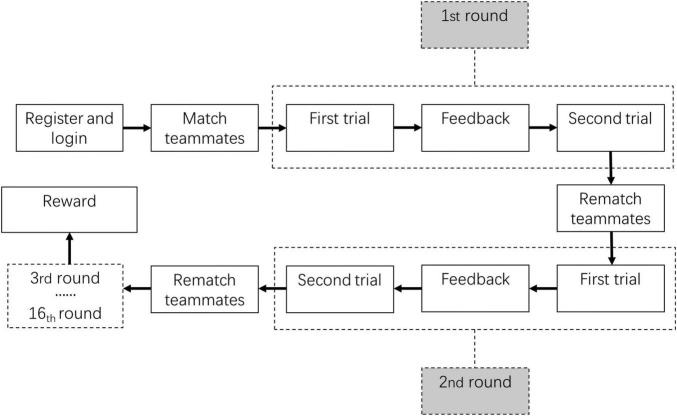
Experimental flow of Study 1a.

Third, each participant was required to perform 16 rounds of allocation tasks, and each round included 2 trials. In each trial, the participants participated in the allocation task, as shown in Figure 1. The only difference was that the team members were fixed during the 2 trials of each round and then re-matched when a new round started. In the interval between the first trial and second trial, each group member observed the allocation decisions of other group members and was informed of RPs allocated to their own individual pool and the shared public pool (i.e., the feedback shown in Figure 2). In the interval between the second trial and a new round, there was no feedback given. The participants were told before the experiment that they could receive the sum of the remaining RPs of four random trials provided that either the individual target or the collective target was reached. One RP was equivalent to 0.1 yuan (see Figure 2 as an illustration). The whole process was achieved by Java.

The explanations of the relative items are listed in Table 1. In the case of one virtual subject’s investment in the public pool of more than 20 RPs, the individual generous amount = public pool input of the virtual subject – 20 RPs (i.e., the fair share that one group member needs to contribute to reach the collective target). In the case of all five virtual subjects’ total investment in the public pool of more than 100 RPs, the generous amount = virtual subjects’ total input – 100 RPs (i.e., five times the fair share). In the case of one virtual subject’s investment in the public pool of less than 20 RPs, the individual self-interest amount = 20 RPs (i.e., the fair share that one group member needs to contribute to reach the collective target) – the public pool input of the virtual subject. In the case of all 5 virtual subjects’ total investment in the public pool being less than 100, the self-interest amount = 100 RPs (i.e., five times the fair share) – the virtual subjects’ total input (see Table 1).

**TABLE 1:** Explanation of relative items (in italics).

Cases	Definition
Public pool input of a virtual subject > 20 RPs	*Individual generous amount* = public pool input of the virtual subject – 20 RPs
Virtual subjects’ total input > 100 RPs	*Generous amount* = virtual subjects’ total input – 100 RPs
Public pool input of a virtual subject < 20 RPs	*Individual self-interest amount* = 20 RPs - public pool input of the virtual subject
Virtual subjects’ total input < 100 RPs	*Self-interest amount* = 100 RPs - virtual subjects’ total input

*The number “20” was the fair share that one group member needed to contribute to reach the collective target. The number “100” was five times the fair share, which was the total public pool input if all virtual subjects contributed the fair share.*

The participants observed the strategies that their teammates took in the first trial to decide which strategy to take in the second trial. The independent variable of the experiment was the allocation data of the virtual subjects in the first trial, while the dependent variable was the allocation data of the participant in the second trial. Study 1a included two independent single-factor four-level within-subject designs (see Table 2). In Table 2, rounds 1 to 4 aimed to explore the impact of the altruistic tendency of one of the partners on people’s propensity to cooperate. In the first trial of these four rounds, one of the five virtual subjects allocated 24, 27, 30, and 33 RPs to the public pool (i.e., the individual generous amounts were 4, 7, 10, 13, respectively), thereby observing the decisions made by the participant regarding the conditions of different individual generous amounts. In Table 2, rounds 5 to 8 aimed to explore the impact of the self-interest tendency of one of the partners on people’s cooperative decision-making. In the first trial of these four rounds, one of the five virtual subjects allocated 18, 15, 12, and 9 RPs to the public pool (i.e., the individual self-interest amounts were 2, 5, 8, and 11, respectively), thereby observing the decisions made by the participant regarding the conditions of different individual self-interest amounts. Rounds 9 and 10 in Table 2 aimed to test whether the participants were more sensitive to the altruism tendency or the self-interest tendency. Round 9 and round 10 utilized a single-factor, two-level in-subject design. Round 9 was the target round in which two virtual subjects invested 24 RPs in the public pool, two virtual subjects invested 16 RPs in the public pool, and one virtual subject invested 20 RPs in the public pool. Round 10 was the control round in which all five virtual subjects invested 20 RPs in the public pool during the first trial. In the first trial of rounds 9 and 10, the total amount of the virtual subjects’ input toward the public pool was equivalent at 100 RPs. We hypothesized that if the participants were more sensitive to the self-interest factor, they would show a greater cooperation tendency in round 10 than in round 9. The virtual subjects who were not mentioned above all contributed 20 RPs to the public pool. All the virtual subjects only chose one pool in which to invest their RPs (see Table 2).

**TABLE 2:** Virtual subjects’ allocation data of the first trial and participants’ solution of the second trial.

Rounds		Virtual subjects’ input					The number of participants taking collective solution	The number of participants taking individual solution	Total	Proportion of collective solution	Proportion of individual solution
		①	②	③	④	⑤					
	Individual generous amount										
1	4	24	20	20	20	20	32	16	48	0.67	0.33
2	7	27	20	20	20	20	31	17	48	0.65	0.35
3	10	30	20	20	20	20	33	15	48	0.69	0.31
4	13	33	20	20	20	20	34	14	48	0.71	0.29
	Individual self-interest amount										
5	2	18	20	20	20	20	26	22	48	0.54	0.36
6	5	15	20	20	20	20	20	28	48	0.42	0.58
7	8	12	20	20	20	20	16	32	48	0.33	0.67
8	11	9	20	20	20	20	15	33	48	0.31	0.69
Comparing sensitivity rounds											
9		24	24	16	16	20	23	25	48	0.48	0.52
10		20	20	20	20	20	35	13	48	0.73	0.27
Extra rounds							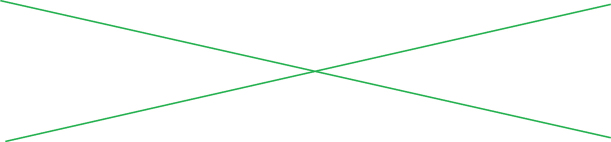				
11		24	20	0	0	0										
12		27	20	0	0	0										
13		20	0	0	0	0										
14		23	22	22	20	0										
15		28	21	20	0	0										
16		24	21	20	20	0										

*Virtual subjects’ allocation data as the independent variable are shown in blue, and participants’ solutions of the second trial as the dependent variable are shown in green. Virtual subjects’ input was the amount of RP virtual subjects invested in the public pool. A total of 48 participants participated in Study 1a.*

According to previous research, the subjects in real online interactive experiments present a proportion of the number of individual strategies, i.e., the number of free-rider strategies: the number of cooperative strategies: the number of altruistic strategies ≈ 2:3:1:4 (Gross and De Dreu, 2019). The free-rider strategy is trying to achieve neither the collective target nor the individual target. The cooperative strategy contributes a fair share to the collective target. The altruistic strategy contributes more than the fair share. To convince the participants of the authenticity of the online experiment and make the number of virtual subjects tend to meet the above proportion, 6 extra rounds were added to the experiment to create a balance (see Table 2). In Study 1a, the sequence of 16 rounds was designed according to the Latin square.

### Results and Discussion

**FIGURE 3:**
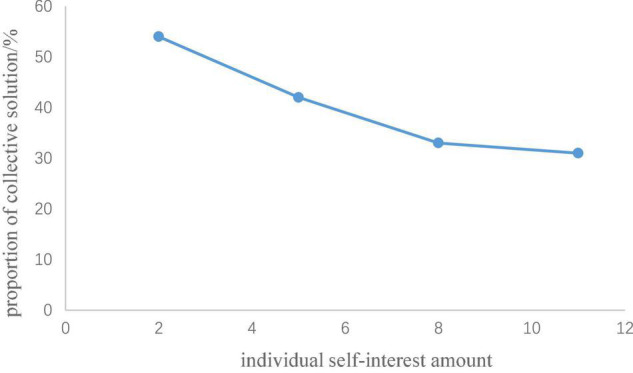
Proportion of collective solution in relationship with individual self-interest amount.

The analysis process was achieved by Python. In Study 1a, the number of participants who chose the collective solution (public pool input > 0, individual pool input = 0) and the individual solution (public pool input = 0, individual pool input = 35) in the second trial of rounds 1 to 8 were counted. Rounds 1 to 4 of the generous investment yielded no significant difference for the proportion of participants who chose a collective solution in the second trial. In rounds 5 to 8, the proportion of participants who chose the collective solution in the second trial showed a downward trend in relation to the increase of the individual self-interest amount (see Table 2 and Figure 3). A matched pair chi-square test was carried out between rounds 5 and 8 in pairs (i.e., a matched pair chi-square test was conducted between rounds 5 and 6, rounds 5 and 7, rounds 5 and 8, rounds 6 and 7, rounds 6 and 8, and rounds 7 and 8). The results showed that the participants were more inclined to choose the collective solution in the round in which the individual self-interest amount was 2 RPs than in the round in which the individual self-interest amount was 8 RPs, χ^2^(1) = 5.06, *p* = 0.021. In addition, the participants were more inclined to choose the collective solution in the rounds in which the individual self-interest amount was 2 RPs than in the round in which the individual self-interest amount was 11 RPs, χ^2^(1) = 4.76, *p* = 0.027. Under the cooperation-oriented situation of Study 1a, from Figure 3 we can see the cooperative tendency of the participants decreased with an increase in the individual self-interest amount but when the individual self-interest amount reached 40% (i.e., the individual self-interest amount was 8 RPs) of the cost of collective solution, the proportion of participants who chose collective solution showed a trend to stabilize.

The matched pair chi-square test of rounds 9 and 10 showed that the participants were more inclined to choose the collective solution in the round in which every group member contributed their fair share than they were in the round in which a generous tendency and equal self-interest tendency coexisted, χ^2^(1) = 4.65, *p* = 0.029. People were more sensitive to self-interest factors that were not conducive to cooperation.

Under the cooperation-oriented situation of Study 1a, the proportion of subjects who chose the collective solution in rounds 1 to 4 showed a ceiling effect. The cooperative tendency in rounds 1 to 4 was too high to detect any significant difference. Therefore, we designed Study 1b under an “individual-oriented” situation.

## Study 1B

In Study 1b, the cost ratio of individually versus collectively solving the shared problem was set as 1.35, which was the “individual-oriented” situation.

Study 1b aimed to explore how the altruism of one partner influences people’s cooperative tendency under an individual-oriented situation when an individual solution is available.

### Method

#### Participants

*A priori* power analysis, carried out using G*Power software (Faul et al., 2007), indicated that to detect a medium-effect size of *d* = 0.5, for the planned χ*^2^* test, with an alpha of 0.05 and power = 0.80, a sample of 48 participants would be needed. Fifty-one undergraduate students from a major public university participated in Study 1b. Data from 2 participants were removed due to duplicated response IDs, and data from an additional 1 participant were removed for questioning the authenticity of the online experiment. This left us with a final sample of 48 participants (*M*_*age*_ = 20.10, *SD* = 1.11, 52% female).

#### Procedure

##### Procedure Part 1

The first step for recruiting participants was the same as in Study 1a.

##### Procedure Part 2

In the second step, the ratio of the individual solution versus the collective solution was adjusted to 1.35. Under the individual-oriented situation of Study 1b, a participant would keep the remaining resources not invested if the participant allocated enough resources to her individual pool to reach a predefined individual target (26 RPs). She would also keep her remaining resources if the group collectively allocated enough resources to the public pool to reach a predefined public target (120 RPs). If the group members did not reach either their private or the public target, they lost everything. Under the individual-oriented condition, if each person invested 26 RPs in the individual pool, they could obtain the remaining 24 RPs. However, if the cooperative method was adopted to achieve the public target, each person also needed to invest 20 RPs and could obtain the remaining 30 RPs. The cost ratio of individual solutions versus collective solutions was 26:20, and the profit ratio of individual solutions versus collective solutions was 24:30. According to the results of previous studies (Gross and De Dreu, 2019), participants tend to adopt the individual solution in this case.

##### Procedure Part 3

Third, each subject was required to perform 16 rounds of allocation tasks, and each round included 2 trials. Study 1b included two single-factor four-level in-subject designs (see Table 3). In Table 3, rounds 1 to 4 explored the impact of different altruism tendencies of one of the partners on people’s propensity to cooperate. In the first trial of these four rounds, one of the five virtual subjects input 22, 24, 26, and 28 RPs into the public pool (i.e., the individual generous amounts were 2, 4, 6, and 8, respectively), thereby observing the decisions made by the subjects in the second trial under the conditions of different individual generous amounts. In Table 3, rounds 5 to 8 were used to explore the impact of self-interest on people’s decision-making. In the first trial of these four rounds, one of the five virtual subjects input 19, 18, 17, and 16 RPs into the public pool (i.e., the individual self-interest amounts were 1, 2, 3, and 4, respectively), thereby observing the decisions made by subjects in the second trial under the conditions of different individual self-interest amounts. Round 9 and round 10 in Table 3 were the same as those described in Study 1a. For the same reason as that stated in Study 1a, we added 6 extra rounds in Study 1b. The virtual subjects who were not mentioned above all contributed 20 RPs to the public pool. All the virtual subjects only chose one pool in which to invest their RPs (see Table 3).

**TABLE 3:** Virtual subjects’ allocation data of the first trial and participants’ solution of the second trial.

Rounds		Virtual subjects’ input					The number of participants taking collective solution	The number of participants taking individual solution	Total	Proportion of collective solution	Proportion of individual solution
		①	②	③	④	⑤					
	Individual generous amount										
1	2	22	20	20	20	20	16	32	48	0.33	0.67
2	4	24	20	20	20	20	19	29	48	0.4	0.6
3	6	26	20	20	20	20	29	19	48	0.6	0.4
4	8	28	20	20	20	20	29	19	48	0.6	0.4
	Individual self-interest amount										
5	1	19	20	20	20	20	11	37	48	0.23	0.77
6	2	18	20	20	20	20	10	38	48	0.21	0.79
7	3	17	20	20	20	20	7	41	48	0.15	0.85
8	4	16	20	20	20	20	3	45	48	0.06	0.94
Comparing sensitivity rounds											
9		24	24	16	16	20	13	35	48	0.27	0.73
10		20	20	20	20	20	22	26	48	0.46	0.54
Extra rounds							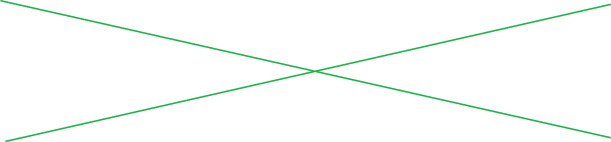				
11		24	20	0	0	0										
12		26	20	0	0	0										
13		20	0	0	0	0										
14		23	22	22	20	0										
15		28	21	20	0	0										
16		24	21	20	20	0										

*Virtual subjects’ allocation data as the independent variable are shown in blue, and participants’ solutions of the second trial as the dependent variable are shown in green. Virtual subjects’ input was the amount of RP virtual subjects invested in the public pool. A total of 48 participants participated in Study 1b.*

### Results and Discussion

**FIGURE 4:**
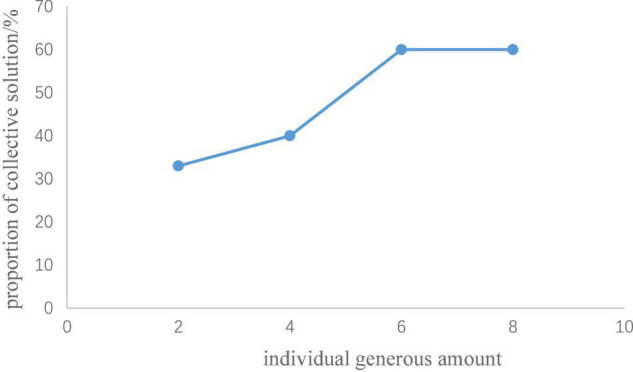
Proportion of collective solution in relationship with individual generous amount.

The analysis process was achieved by using Python. In Study 1b, the number of subjects who chose the collective solution (public pool input > 0, individual pool input = 0) and the individual solution (public pool input = 0, individual pool input = 35) in the second trials of rounds 1 to 8 were counted. In rounds 5 to 8, the number of subjects who chose the individual solution in the second trial yielded no significant difference. In rounds 1 to 4, the proportion of people who chose the collective solution in the second trial showed an upward trend in relation with an increase in the individual generous amount (see Table 3 and Figure 4). A matched pair chi-square test was carried out between rounds 1 and 4 in pairs (i.e., a matched pair chi-square test was conducted between rounds 1 and 2, rounds 1 and 3, rounds 1 and 4, rounds 2 and 3, rounds 2 and 4, and rounds 3 and 4). The results showed that participants were more inclined to choose the collective solution in the round in which the individual generous amount was 6 RPs than they were in the round in which the individual generous amount was 2 RPs, χ^2^(1) = 6.04, *p* = 0.013. The participants were more inclined to choose a collective solution in the round in which the individual generous amount was 8 RPs than they were in the round in which the individual generous amount was 2, χ^2^(1) = 5.63, *p* = 0.016. Under the individual-oriented situation of Study 1b, from Figure 4 we can see the cooperative tendency of the participants increased with an increase in the individual generous amount but when the individual generous amount reached 30% (i.e., the individual self-interest amount was 8 RPs) of the cost of collective solution, the proportion of participants who chose collective solution showed a trend to stabilize.

The matched pair Chi-square test of rounds 9 and 10 of Table 3 showed that the participants were more inclined to choose the collective solution in the rounds in which every group member contributed their fair share than in those in which the generous tendency and an equal self-interest tendency existed, χ^2^(1) = 2.78, *p* = 0.093.

## Study 2A

After we prove one group member’s behavior can influence participants’ individual or collective strategy decision-making in Study 1a and Study 1b, we intended to investigate the exact nature of the relationship between others’ and our own behavior in a detailed way.

Study 2a aimed to explore whether people pay more attention to the number of altruists or are more likely to be influenced by a few obvious altruists. In this study, we distributed a generous amount of 10 RPs (i.e., see Table 1 for the definition of “a generous amount”) equally among 2 to 5 virtual subjects in the group to explore whether there was a difference in people’s trust in the a collective solution under the convergent (i.e., a generous amount of 10 RPs was equally allocated to 2 virtual subjects) or divergent (i.e., a generous amount of 10 RPs was equally allocated to 5 virtual subjects) distribution of an equivalent generous amount.

### Method

#### Participants

*A priori* power analysis, carried out using G*Power software (Faul et al., 2007), indicated that to detect a medium-effect size of *d* = 0.5, for the planned T test, with an alpha of 0.05 and power = 0.80, a sample of 34 participants would be needed. Seventy-three college students from a university took part in Study 2a. We obtained 68 valid data points (*M*_*age*_ = 19.32, *SD* = 1.21, 58.8% females) after excluding 2 subjects who did not understand the rules well and 3 subjects who questioned the authenticity of the online experiments. The sample size provided enough sensitivity to detect a minimum effect size of Cohen’s *d* = 0.44 (Cohen, 1992), with the power set at 0.80 and the alpha value at 0.05.

#### Procedure

The procedure of Study 2a was the same as that used in Study 1a. Study 2a adopted a single-factor, four-level, in-test design consisting of 10 rounds of tasks (see Table 4). In Table 4, in rounds 1 to 4, a generous amount of 10 RPs was distributed equally to 2–5 virtual subjects. For the same reason as that explained in Study 1a, six extra rounds were added to Study 2a. The virtual subjects who were not mentioned above all contributed 20 RPs to the public pool. All the virtual subjects only chose one pool in which to invest their RPs (see Table 4). The position of the six extra rounds was fixed, and rounds 1 to 4 were embedded in the extra rounds according to Latin square order (see the experimental material file in detail).

**TABLE 4:** Virtual subjects’ allocation data of the first trial in Study 2a.

Rounds		Virtual subjects				
		①	②	③	④	⑤
	Generous amount					
1	10	22	22	22	22	22
2	10	23	23	22	22	20
3	10	24	23	23	20	20
4	10	25	25	20	20	20
5		20	23	20	15	0
6		25	20	18	0	0
7		20	22	0	0	0
8		25	25	20	0	0
9		24	22	20	15	0
10		30	20	20	20	0

*Virtual subjects’ input was the amount of RPs the virtual subjects invested in the public pool. A total of 68 participants participated in Study 2a.*

### Analysis

**FIGURE 5:**
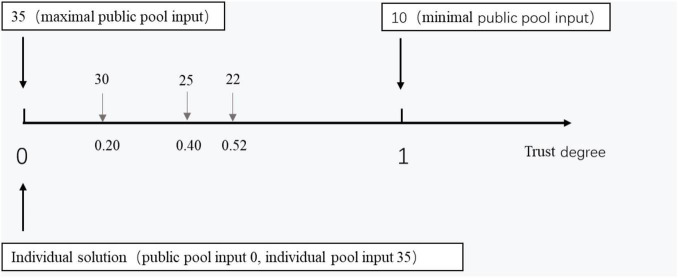
Relationship between public pool input and trust degree. Trust degree = 1 – (public pool input – minimal public pool input)/(maximal public pool input – minimal public pool input).

The analysis process was achieved by using Python. Compared to Study 1a and Study 1b, the manipulation effect was less significant in Study 2a (i.e., the matched pair chi-square test was not significant). Therefore, we converted the dependent variables to continuous variables to perform further analysis. When the participants chose the collective solution, the public pool input and the degree of trust in collective solution met the following:

Trust degree = 1 − (public pool input – minimal public pool input)/(maximal public pool input – minimal public pool input) (see Figure 5 as an illustration). The trust degree increased as a function of a decrease in the public pool input. When the public pool input was at the maximum, the trust degree was at the minimum; furthermore, when the public pool input was minimal, the trust degree was maximal. We also defined the individual solution as a trust degree of “0”. The minimal public pool input referred to the minimal public pool input of all the subjects’ trials in Study 2a, while the maximal public pool input referred to the maximal public pool input of all the subjects’ trials in Study 2a.

**FIGURE 6:**
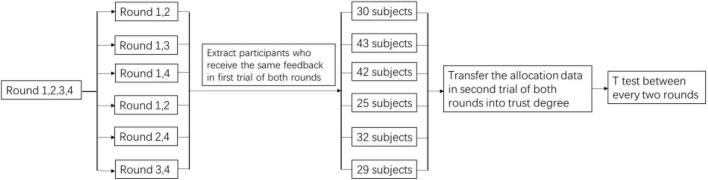
Analysis procedure.

The allocation data of the second trial in rounds 1 to 4 of Table 4 were converted into trust degrees according to the above rules. Considering that the public pool input for the first trial of each round was uncertain, we could not control whether the participant reached the collective target in the first trial, which would definitely affect the decision of the second trial. Therefore, we treated whether the public target was reached in the first trial as a covariate. Taking round 1 and round 4 as an example, the subjects who received the same feedback (i.e., either the subjects reached the collective target in the first trial of both rounds, or the subjects did not reach the collective target in the first trial of both rounds) in the first trial of round 1 and round 4 were extracted. Then, the second trial input data of these two rounds was converted into trust degrees; finally, the trust degrees from the second trial of round 1 and round 4 were tested by a T test. The trust degrees from the second trial of rounds 1 to 4 were tested by T tests in pairs (i.e., there was a T test conducted between rounds 1 and 2, rounds 1 and 3, rounds 1 and 4, rounds 2 and 3, rounds 2 and 4, and rounds 3 and 4) (see Figure 6 as an illustration).

### Results and Discussion

**FIGURE 7:**
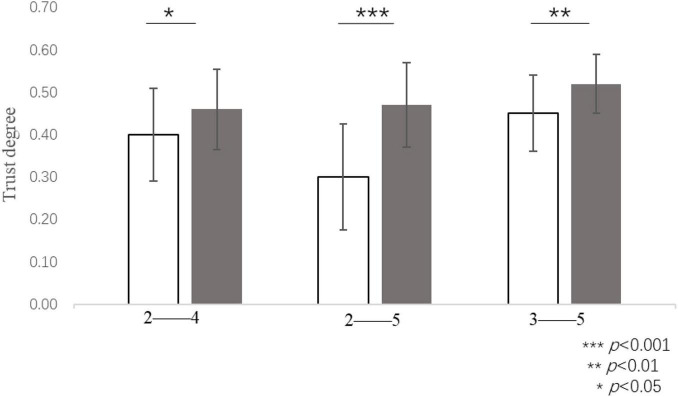
Trust degree when equal generous amounts were allocated to different numbers of group members, e.g.: 2–4 reflects both the round in which a generous amount of 10 RPs was allocated to 2 virtual subjects and the round in which a generous amount of 10 RPs was allocated to 4 virtual subjects, respectively. ***P < 0.001, **P < 0.01, *P < 0.05.

The trust degree in the round of four virtual subjects who were assigned a generous amount of 10 RPs (*M* = 0.46, *SD* = 0.19) was significantly higher than that of two virtual subjects who were assigned a generous amount of 10 RPs [*M* = 0.40, *SD* = 0.22, *t*(*31*) = 2.27, *p* = 0.030, *d* = 0.40]. The participants’ trust degree in the round including five virtual subjects who were assigned a generous amount of 10 RPs (*M* = 0.52, *SD* = 0.14) was significantly higher than that of three virtual subjects who were assigned the same generous amount [*M* = 0.45, *SD* = 0.18, *t*(*42*) = 3.75, *p* = 0.001, *d* = 0.57]. The participants’ trust degree in the round including five virtual subjects who were assigned a generous amount of 10 RPs (*M* = 0.47, *SD* = 0.20) was significantly higher than that of two virtual subjects who were assigned an equal generous amount [*M* = 0.30, *SD* = 0.25, *t*(*41*) = 4.19, *p* < 0.001, *d* = 0.65] (see Figure 7). Excluding the participants who had not been extracted as the target data, 65 participants contributed to the final data, thereby accounting for 95.6% of all the participants in Study 2a. Overall, the difference between adjacent rounds was not significant. The experimental results showed that an equal generous amount was more conducive to cooperation in the form of a divergent distribution. The cooperation tendency of the participants was stronger when an equal generous amount was distributed to more people on the team.

## Study 2B

Study 2b aimed to explore whether people pay more attention to the number of teammates who are self-interested or are more likely to be influenced by a few obvious self-interested teammates. Study 2b distributed a self-interest-based amount of 5 RPs equally among 2–5 virtual subjects in the team to explore whether there was a difference in people’s trust degree under the convergent (i.e., the self-interest amount of 5 RPs was equally allocated to two virtual subjects) or divergent (i.e., the self-interest amount of 5 RPs was equally allocated to five virtual subjects) distribution of an equivalent self-interest-based amount.

### Method

#### Participants

*A priori* power analysis, which was carried out using G*Power software (Faul et al., 2007), indicated that to detect a medium-effect size of *f* = 0.25 for the planned F test, with an alpha value of 0.05 and a power of 0.80, a sample of 33 participants would be needed. A total of 110 college students took part in the experiment. We obtained 104 valid data points (*M*_*age*_ = 19.88, *SD* = 1.50, 67.3% females), excluding 2 subjects who did not understand the rules well and 4 subjects who questioned the authenticity of the online experiments. The sample size provided sensitivity to detect a minimum effect size of Cohen’s *d* = 0.36, with power set at 0.80 and an alpha value of 0.05.

#### Procedure

The procedure of Study 2b was the same as that used in Study 2a. Study 2b adopted a single-factor, four-level, in-test design consisting of 10 rounds of tasks (see Table 5). As seen in Table 5, in the first trial of rounds 1 to 4, a self-interest-based amount of 5 RPs was distributed equally to 2–5 subjects. For the same reason as that described in Study 1a, six extra rounds were added. The virtual subjects who were not mentioned above all contributed 20 RPs to the public pool. All the virtual subjects only chose one pool in which to invest their RPs (see Table 5). The position of the six extra rounds was fixed, and rounds 1 to 4 were embedded in the extra rounds according to Latin square order (see the experimental material file in detail).

**TABLE 5:** Virtual subjects’ allocation data of the first trial in Study 2b.

Rounds		Virtual subjects				
		①	②	③	④	⑤
	Self-interest amount					
1	5	19	19	19	19	19
2	5	19	19	19	18	20
3	5	19	18	18	20	20
4	5	18	17	20	20	20
Extra rounds						
5		30	25	25	25	0
6		25	25	0	0	0
7		28	25	25	0	0
8		30	25	25	20	15
9		24	23	0	0	0
10		25	25	25	20	0

*Virtual subjects’ input was the amount of RPs that the virtual subjects invested in the public pool. A total of 104 participants participated in Study 2b.*

### Results and Discussion

**FIGURE 8:**
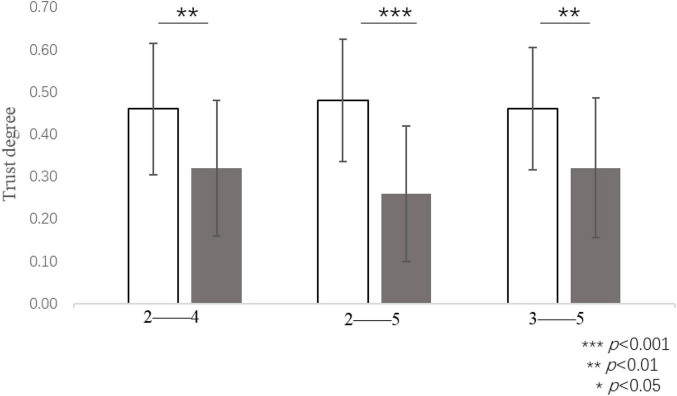
Trust degree when equal self-interest-based amounts were allocated to different numbers of group members, e.g.: 2–4 refers to both the round in which a self-interest-based amount of 5 RPs was assigned to 2 virtual subjects and the round in which a self-interest-based amount of 5 RPs was assigned to 4 virtual subjects, respectively. ***P < 0.001, **P < 0.01.

Study 2b adopted the same Python-based analysis method as that used in Study 2a. The trust degree of the participants in the round of two virtual subjects who were assigned a self-interest-based amount of 5 RPs (*M* = 0.46, *SD* = 0.31) was significantly higher than that of four virtual subjects who were assigned a self-interest-based amount of 5 RPs [*M* = 0.32, *SD* = 0.32, *t*(*55*) = 2.91, *p* = 0.005, *d* = 0.39]. The participants’ trust degree in the round of three virtual subjects who were assigned a self-interest-based amount of 5 RPs (*M* = 0.46, *SD* = 0.29) was significantly higher than that of five virtual subjects who were assigned a self-interest amount of 5 RPs [*M* = 0.32, *SD* = 0.33, *t*(*63*) = 3.64, *p* = 0.001, *d* = 0.45]. The participants’ trust degree in the round of two virtual subjects who were assigned a self-interest-based amount of 5 RPs (*M* = 0.48, *SD* = 0.29) was significantly higher than that of five virtual subjects who were assigned a self-interest-based amount of 5 RPs [*M* = 0.26, *SD* = 0.32, *t*(*46*) = 4.08, *p* < 0.001, *d* = 0.59] (see Figure 8). Excluding the participants who had not been extracted as the target data, 95 participants contributed to the final data, thereby accounting for 91.3% of all participants in Study 2b. Overall, the difference between adjacent rounds was not significant. The results showed that an equal self-interest-based amount was more conducive to cooperation in the form of convergent contribution and that the cooperative tendency of participants was weaker when an equal self-interest-based amount was distributed to more people on the team.

## General Discussion

The results are consistent with our hypothesis. The results of Study 1a and Study 1b not only showed that participants’ decision-making was affected by their teammates’ tendency toward self-interest and altruism but also presented an upper limit of the influence of their teammates’ credibility. The reason for this outcome may be that once the teammates’ credibility exceeded a certain amount, the cost ratio of the individual solution versus the collective solution mattered more in participants’ decision-making. The results of Study 2a and Study 2b showed that, with the total altruistic/self-interest tendency unchanged, the participants attached more importance to the number of team members who presented a slightly altruistic or self-interest-based tendency rather than the generously altruistic team members or the extremely self-interested team members. The participants might have thought that the proportion of their teammates who were willing to cooperate in a team could better predict the possibility of cooperation success in the second trial.

Our study found that the divergent distribution of generosity can improve the cooperative tendency of the subjects, which has some implications for people’s preference for fairness. Chou et al. (2016) demonstrated that the monomorphic society of fair preference would be the only evolutionarily stable equilibrium by establishing evolutionary game models and random evolution simulation models. Humans’ preference for fairness may result from the adaptive advantage of instinctive fairness behavior in early human evolution (Chen et al., 2020; Iwamura et al., 2020). Cooperation is a necessary and more effective means for human society to increase individual welfare; however, people who are in the search for cooperation need to face the instability of cooperation opportunities and benefits. Although people can obtain the maximum profit during one-shot cooperation when focusing on the benefit of cooperation, it will become more difficult for these individuals to find such partners in the future. Thus, their opportunities for cooperation will decrease in the long term. In contrast, although it will increase their chance of cooperation when people contribute much more than their fair share to the collective solution, the profit attached to one-shot cooperation decreases. Regardless of whether cooperation opportunities decrease or the cooperation benefits decline, this approach is not conducive to an individual’s survival. As a result, a fair state of cooperation is also preferred from the individual’s point of view. The group in which every member both contributes and benefits equally tends to convince participants that the group can sustain effective cooperation in the long run.

We must acknowledge that the sensitivity difference between the generous factor and the self-interest factor in Study 1a can also be explained by people’s preference for fairness. Participants had a higher tendency to cooperate in the control round, which may be because the subjects preferred a fair state in which everyone contributed equally to the collective target from the overall perspective. From a local point of view (i.e., the participants paid independent attention to the generous factor and the self-interest factor), however, this may be because the subjects were indeed more sensitive to the self-interest factor when generous and self-interest factors coexisted. In either way, this outcome reflects the fact that when individual solutions are available, people’s propensity to collaborate increases when all partners tend to cooperate to a similar extent. Further research needs to be done to explore the sensitivity difference.

Overall, the results indicated that the participants showed largely rational responses. Except for a few participants who did not understand the experimental rules thoroughly, there was no one to invest RPs in either the public pool or private pool. Because only one of the individual targets and the collective target needed to be reached, if the participants invested RPs in both pools, then the remaining RPs would decrease dramatically. Even if there were several participants investing in both pools during the first or second round, after observing his or her teammates’ behavior, he or she would quickly switch to the one-pool strategy (i.e., considering that there were only several rounds of two-pool investing, in the analysis process, the larger number of the two invested values was regarded as the participants’ real decision). Moreover, the variables we manipulated reasonably affected the participants’ probability of contributing to the collective solution. Any sort of variability in the contributions of others would suggest that their contributions were somewhat unpredictable, thus creating a risk that one’s own contribution would be wasted and thereby changing one’s own decision-making. Thus, we must ask the following question: if everyone was simply rational and insisted on the collective solution regardless of any sort of variability, would the Nash equilibrium (i.e., everyone can obtain the maximal profit) (Holler and Klose-Ullmann, 2020) be achieved? Obviously, the “if” is impossible to determine. It should be emphasized that since one participant must know the others’ strategies to know his or her own feasible strategy set, the others cannot determine their feasible strategies without knowing the one participant’s strategy (Ye and Hu, 2017; Yu et al., 2017; Rubinstein, 2018; Holler and Klose-Ullmann, 2020). In a real interactive game that used the same paradigm as that used in our study, Gross and De Dreu (2019) observed that few people attempted to achieve the Nash equilibrium by insisting on the collective solution. More specifically, there is literature (Tyran, 2004) on step-level games such as ours in which it is argued that the rational strategy is to contribute more when the probability of being “pivotal” is higher. Interestingly, there seems to be a “bandwagon” effect beyond this point, such that people contribute even when their contribution is not needed. These results all indicate that people cannot be completely rational, i.e., similar to machines, and that the Nash equilibrium is impossible to achieve in these circumstances.

As material resources become increasingly abundant, human individualism is becoming more abundant (Santos et al., 2017). An increasing number of individual solutions to shared problems have emerged (Izquierdo et al., 2010). As a result, it is worthwhile for us to conduct more research to understand how individual solutions to shared problems influence the human inclination for cooperation and coordination. In future research, we plan to design more delicate experiments to explore the sensitivity difference between the generous factor and the self-interest-based factor. The explicit reason why the divergent distribution of generous amounts and the convergent distribution of self-interest-based amounts are preferred is also worthwhile to determine. Moreover, the interaction between the cost ratio and the teammates’ credibility may be a practical tissue worth examining. If the strong Nash equilibrium (i.e., solutions for a non-cooperative environment where players are allowed to discuss their strategies and to form coalitions without any binding commitment) (Petrosyan et al., 2017) was utilized when the individual solution was available, would the Nash equilibrium be achieved or would cooperation at least be facilitated?

## Data Availability Statement

The original contributions presented in the study are included in the article/Supplementary Material, further inquiries can be directed to the corresponding author.

## Ethics Statement

The studies involving human participants were reviewed and approved by South China Normal University. The patients/participants provided their written informed consent to participate in this study.

## Author Contributions

MZ developed the study concept and drafted the manuscript. JF performed testing and data collection. YY, JJL, JBL, and LM did the proofreading. NQ, JBL, and JDD provided critical revisions and participated in the final revision of the manuscript. All authors approved the final version of the manuscript for submission.

## Conflict of Interest

The authors declare that the research was conducted in the absence of any commercial or financial relationships that could be construed as a potential conflict of interest.

## Publisher’s Note

All claims expressed in this article are solely those of the authors and do not necessarily represent those of their affiliated organizations, or those of the publisher, the editors and the reviewers. Any product that may be evaluated in this article, or claim that may be made by its manufacturer, is not guaranteed or endorsed by the publisher.
